# Global burden of injuries: it is time to understand the data in order to intervene

**DOI:** 10.1136/injuryprev-2020-043937

**Published:** 2020-10-01

**Authors:** Patricia Jannet Garcia

**Affiliations:** School of Public Health, Universidad Peruana Cayetano Heredia, Lima, Peru

**Keywords:** burden of disease, epidemiology, global

The field of population health has benefited in recent years due to greater visibility in the popular domain as well as advances in the underlying data and methods. However, injury burden has not always received exposure and attention equitable to its global importance. This is surprising given the field of injury prevention is to some extent predicated on the principle that exposure to injury risk is universal. Injuries such as falls are widely relatable. Among people who have an injury from a fall, though, some may survive unscathed and some may develop permanent disability or die. The accompanying studies in this series on the global burden of injury are an effort to investigate and understand these trends across injuries on broader levels.

Injuries and their burden can be reduced with effective measures of prevention and treatment. However, more nuanced measurement of injury risk and burden is critical for adopting more widespread prevention and treatment measures. When it comes to rates of different injuries, questions such as why, how, where, what, when, and so on… are key questions that these series are addressing in order to improve our knowledge and inform our response.

In this series, the researchers frame a number of compelling questions that are germane to dialogue on injury prevention but always require a local perspective. Why did the age-standardised incidence of fire-related injuries vary 20-fold in 2017 across countries?^1^ How does the age-standardised mortality-to-incidence ratio vary 25-fold between Oceania and Australasia? Why is mortality from road injuries decreasing in all global regions except South Asia and Southern Latin America?^2^ How much do disability rates from falls vary depending on region of the world?^3^ The disparities between countries and regions can be seen on a global scale in this series, but the most critical insight and solutions must be investigated and implemented on a local scale.

The Global Burden of Disease (GBD) Study framework used across these studies is empowering for researchers and policy makers who are positioned to investigate and intervene on these issues. Detailed fatal and non-fatal burden estimates by age, sex, year, location, cause of injury and nature of injury have all been made publicly available, though the results reported in this series represent a small fraction of all estimates in the annual GBD series.^4–6^ These studies and the GBD estimates should, in short, be used to make the world a safer place and to decrease inequities between regions. By documenting the burden and its magnitude and its trend, we will have a foundation and a call for investment in safe infrastructure, laws and regulations pertaining to road safety, alcohol prevention strategies, ensuring access to medical care, and various other avenues should continue to be pressing priorities for governments and healthcare systems.

However, the ability to act on data is also only as powerful as the data are accurate. While the GBD Study has successfully developed many novel methodologies in a robust modelling framework, a common theme among each study in this series is the need for more data to inform this estimation process. The data sources must be improved and GBD shows clearly where the holes are through its estimation of uncertainty. These series call for better injury surveillance data and additional sources of administrative clinical data, and new data collection systems for injuries should all be integrated into future updates in population health measurement. An organised approach to data collection and processing allows for leveraging of scientific processes in refinement for the GBD Study, and facilitates benchmarking and progress tracking, which are critical elements in global dialogue on injury prevention. Therefore, the implications of this research compendium should guide more investment in injury prevention research, implementation and better data, and should serve as a reminder that injury risk is truly global but that there are marked differences between regions so injuries worsen inequities and that we should all feel invested in contributing to making the world a safer place. This series is overwhelming with detailed results and expansive scope but a global, comprehensive perspective should facilitate the kind of insightful dialogue and knowledge-sharing that help frame the scope of the problem and help policy makers and planners develop more focused programmes to decrease future injury burden.
